# Understanding aneurysmal type 1 neovascularization (polypoidal choroidal vasculopathy): a lesson in the taxonomy of ‘expanded spectra’ – a review

**DOI:** 10.1111/ceo.13114

**Published:** 2017-12-26

**Authors:** Kunal K Dansingani, Orly Gal‐Or, Srinivas R Sadda, Lawrence A Yannuzzi, K Bailey Freund

**Affiliations:** ^1^ Department of Ophthalmology, University of Pittsburgh Medical Center Pittsburgh Pennsylvania USA; ^2^ Moorfields Eye Hospital London UK; ^3^ Vitreous Retina Macula Consultants of New York New York New York USA; ^4^ LuEsther T. Mertz Retinal Research Center Manhattan Eye, Ear and Throat Hospital New York New York USA; ^5^ Rabin Medical Center Petah‐Tikva Israel; ^6^ Doheny Eye Institute Los Angeles California USA; ^7^ Department of Ophthalmology David Geffen School of Medicine at University of California Los Angeles Los Angeles California USA

**Keywords:** imaging, macular degeneration, neovascularization, pachychoroid, polypoidal

## Abstract

The term aneurysmal type 1 neovascularization is derived from terminology, which is established in the literature but has fallen out of use. We believe that aneurysmal type 1 neovascularization accurately describes the lesions which define the entity known as polypoidal choroidal vasculopathy (PCV). Over the last three decades, the clinical spectrum of PCV has expanded to recognize the occurrence of the aneurysmal (polypoidal) lesions in different contexts, resulting in a complex and unwieldy taxonomy based sometimes on circumstantial findings rather than mechanistic considerations. Advances in multimodal imaging provides increasingly convincing evidence that the lesions which define various forms of PCV are indeed vascular and arise from type 1 neovascular networks. The understanding of PCV as type 1 neovascularization with aneurysms renews focus on the question as to why some patients with type 1 neovascularization develop aneurysms while others do not. Conceptual themes and potential for further study are discussed.

## Introduction

Aneurysmal type 1 neovascularization is a generic descriptor derived from terminology, which is established in the literature but has fallen out of use. We believe that aneurysmal type 1 neovascularization accurately describes the lesions which define the entity known as polypoidal choroidal vasculopathy (PCV) and its expanded demographic, clinical and contextual spectrum.

The history of our understanding of PCV spans more than three decades, and the terminology and publication record on PCV have grown exponentially over that time. However, the current literature on PCV poses a considerable challenge to those new to the field, such as trainees, fellows and young researchers, who have to grapple with an increasingly complex classification scheme, making PCV difficult to understand and teach.

The objective of this commentary is to review selected PCV literature using its natural chronology to attempt to replicate the process by which colleagues most familiar with the historical contexts surrounding PCV have developed a sound and incrementally acquired understanding of the fundamentals. Some of the concepts and views presented might conflict with strongly held dogmas, but the intention is to provoke discussion of a challenging variety and stimulate a slightly different approach from that which currently prevails.

Although treatment paradigms and response to treatment are beyond the scope of this descriptive review, it should be borne in mind that differential responses to treatment can, at times, influence classification strategies retrospectively.

## Early Descriptions

### The 1980s

The term ‘idiopathic polypoidal choroidal vasculopathy’ was introduced by Yannuzzi *et al*. in 1982 at the annual meeting of the Macula Society, and first appeared in the peer‐reviewed literature in 1990.^1^ A series of 11 patients was described with a degenerative disease featuring exudative and haemorrhagic pigment epithelial and neurosensory detachments. On ophthalmoscopy, the patients exhibited orange lesions, mostly in the *peripapillary* region, and haemorrhagic and exudative neurosensory detachments. Several of the patients were middle‐aged, not elderly (mean 58 years, range 40–71) and six of the 11 patients were of African ancestry.

In the same year, Kleiner *et al*. described eight patients with ‘posterior uveal bleeding syndrome’.^2^ In this series, the median age of patients was 57 (range 40–79) years and seven of the eight patients were of African descent. Interestingly, the same cluster of findings had been noted by Stern *et al*. 5 years earlier, in three female African American patients.^3^

The patients with PCV or posterior uveal bleeding syndrome did not exhibit drusen. Moreover, the character and location of the vascular lesions themselves were unknown, but it was postulated that they arose from the choroidal circulation. Although they were appreciated as possibly representing a form of choroidal neovascularization (CNV), it was noted that the phenotype did not fit into established demographic and aetiological classification schemes for the differential diagnosis of CNV.

In the early 1990s, available reports on neovascular age‐related macular degeneration (AMD) in patients of African ancestry, both in the United Kingdom and in the West Indies, had estimated an incidence of 0.1–0.4%, compared with 3.5% in Caucasian patients over 50 years of age.^4^, ^5^ The rarity of neovascular AMD in these cohorts and their relative predilection for PCV were therefore thought to support the interpretation of PCV as a distinct and newly described entity.

Developments in the understanding of the nature of PCV have been determined to a great extent by developments in imaging. Fluorescein angiography of the posterior segment had been described in the 1960s.^6^ Its application to PCV showed hyperfluorescence and accentuated visualization of the polypoidal lesions and, in two of the series, hyperfluorescence of adjacent features with microvascular morphologies and late leakage suggestive of CNV.^1^, ^2^, ^3^

Some of the early cases were treated with argon or krypton laser photocoagulation, which was shown to be capable of controlling the haemorrhagic and exudative components, with subsequent visual loss attributed to untreatable CNV at the fovea or subfoveal scarring from chronic serosanguineous detachment.^1^

### The 1990s

The early literature described PCV as a peripapillary form of neovascularization, characterized by ‘polypoidal’ vascular lesions or ‘choroidal excrescences’, which were thought to be a form of CNV, and which were overrepresented in middle‐aged African females.^1^ During the 1990s, the description of PCV was consolidated by further case descriptions and by the development of indocyanine green angiography (ICGA). In 1992, Spaide *et al*. published a summary analysis of ICGA performed on 12 patients with PCV in a paper which defined PCV by the presence of (i) a branching vascular network, which became hyperfluorescent early in the angiogram and later evolved into a hyperfluorescent focus (‘plaque’) that masked the choriocapillaris and (ii) the polypoidal structures themselves, which became hyperfluorescent early in the angiogram and subsequently stained or leaked slowly from their walls.^7^ Although ICGA offered better visualization of vascular details deep to the retinal pigment epithelium (RPE), it was still not possible to determine the depth of polypoidal lesions and branching vascular networks with certainty.

In 1994, MacCumber *et al*. studied an enucleated eye from a patient with multiple bilateral serosanguineous detachments of the retina and RPE.^8^ They found extensive fibrovascular proliferation in the subretinal space and on either side of Bruch's membrane. They also noted vascular elements traversing Bruch's membrane but, due to the advanced nature of disease in this eye, could not conclude the exact depth at which pathological processes had started. Importantly, a history of aneurysmal or polypoidal lesions had not been documented in the study eye and it was later reported by Yannuzzi *et al*. that the fellow eye of this patient did not show angiographic features of PCV.^9^ Spraul *et al*. also studied an eye from a 47‐year‐old black female with angiographic PCV, enucleated for angle closure glaucoma following massive subretinal haemorrhage, but they were unable to find histological features to correlate with the angiographic findings.^10^

In 1997, Yannuzzi *et al*. described 20 patients with PCV and discussed sex, race, clinical course and the character and development of the vascular lesions, using colour photographs and ICGA.^9^ The authors noted that, while most cases were peripapillary (with variable extent from the disc), five of the cases had vascular findings confined to the macula. They postulated that the ‘aneurysmal, polypoidal elements’ themselves represented proliferative components at the advancing edge of the vascular network, although this idea could not be supported without longitudinal study. This cohort included four Asian patients. The authors suggested that black patients were at greatest risk compared with white patients and that Asian patients had intermediate risk. They also explained in their discussion that they had recently had the opportunity to peer‐review two independent series of Japanese patients with haemorrhagic maculopathy consistent with idiopathic PCV (from among patients who had previously been classified as having AMD, albeit at a young age and without drusen), and that it had been concluded by the Japanese investigators that PCV is relatively common in Asians and that it can masquerade as neovascular AMD. A year later, Moorthy *et al*. described seven patients of various ethnicities with polypoidal lesions at the macula.^11^

### The 2000s

**Figure 1:**
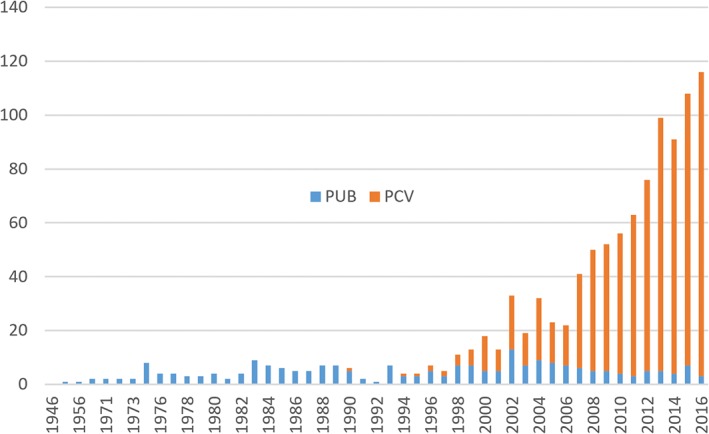
PubMed search results by year for ‘posterior uveal bleeding’ (PUB) and ‘polypoidal choroidal vasculopathy’ (PCV).

The turn of the century saw a dramatic expansion of the literature on PCV with several papers using the ICGA findings of Spaide *et al*. to define and study PCV (Fig. 1).

Lafaut's group published a case report with clinicopathological correlation of an 81‐year‐old male with serosanguineous pigment epithelial detachment (PED) with fibrovascular tissue in one eye and polypoidal lesions detected by ICGA in both eyes. The study eye had undergone surgical extraction of the macular tissue anterior to Bruch's membrane and histology localized the fibrovascular tissue to the external side of basal laminar and basal linear deposits which in turn were immediately external to the RPE. The vascular elements within the fibrovascular tissue were mostly thin‐walled with occasional pericytes, with only one thick‐walled vessel described in the complex. The authors expressed surprise that the aneurysmal lesions correlating with the polypoidal angiographic findings were located in the sub‐RPE space, apparently having arisen from the neovascular tissue and *not* in the inner choroid. Similar anatomical findings were reported by Terasaki *et al*., who extracted fibrovascular tissue during macular translocation surgery on two Japanese patients with PCV, one of whom had also undergone macular radiation therapy for subretinal neovascular membrane.^12^

In 2002, Rosa *et al*. published a case study describing the clinical course and the post‐enucleation findings in a patient with PCV.^13^ Clinically, this patient exhibited features more convincing for idiopathic PCV than those seen previously in MacCumber's case.^8^ Rosa *et al*. noted that the ‘thin‐walled, cavernous vascular channels’ which traversed Bruch's membrane ‘originated from branches of the short posterior ciliary arteries’, a finding which was cited, corroborated and discussed in subsequent histopathological studies of PCV. An example is the light and electron microscopy study conducted by Kuroiwa *et al*., who demonstrated the presence of arterioles and venules beneath and adjacent to Bruch's membrane and noted that the arterioles featured elastic lamellae. They also speculated that these arteriolar elements might account for the pulsatile character often observed during angiography of polypoidal lesions.^14^ The arteriolar character of the vessels comprising the abnormal tissue was also suggested subsequently by Nakashizuka *et al*., who observed hyalinization which they interpreted as analogous to arteriosclerosis.^15^ Definitive conclusions about the anatomical relationship of polypoidal lesions to Bruch's membrane could not be drawn, however, and this may have been because of the advanced disease seen in the enucleated eyes.

In 2005, Yuzawa *et al*. examined 45 eyes in Japanese patients aged 53–81 years with ‘typical PCV’, defined by serosanguineous retinal and/or PEDs and ICGA findings consistent with polypoidal lesions and branching vascular networks. They observed on ICGA that the branching vascular networks overlaid large choroidal vessels in all eyes and exhibited late placoid hyperfluorescence on ICGA in some but not all eyes. They concluded that PCV must be a heterogenous family of three conditions: (i) a primary choroidal vasculopathy, as suggested by Yannuzzi *et al*.; (ii) ‘polypoidal choroidal neovascularization’, as described by Uyama; and (iii) ‘radiation‐induced choroidal neovasculopathy’, after Terasaki *et al*.^1^, ^11^, ^12^, ^16^, ^17^, ^18^ Although Yuzawa *et al*. interpreted their data by showing that PCV in their cohort was represented as a primary choroidal vasculopathy (‘branching vessels do not represent CNV …,’) examination of their figures with the benefit of hindsight and experience with more recent optical coherence tomography (OCT) angiography findings suggests in fact that their patients did indeed have branching vascular networks.^19^

Clinical and histopathological findings in the 1990s and early 2000s showed that polypoidal lesions of PCV are indeed vascular in nature and appear to arise from networks of vessels which, compared with those of neovascular AMD, are more extensive, larger in calibre, thin‐walled and often related to arteriolar vessels in the choroid. However, the expansion of knowledge concerning the clinical spectrum of PCV and the histopathological findings raised as much uncertainty as clarification regarding the exact nature and location of polypoidal lesions and whether PCV was a single entity or whether it merited subclassification.

## Thematic Discussions

### Neovascularization

Angiogenesis is a normal developmental process. Neovascularization refers to angiogenesis in a pathological context and implies that the newly formed blood vessels adopt configurations and occupy locations which *differ* from those of physiological vessels. For example, in ocular ischaemic disease, neovascular complexes arising from the retinal circulation develop and grow along the posterior hyaloid face, whereas anterior segment rubeosis forms over the iris surface and trabecular meshwork. Neovascular tissue is therefore generally *ectopic.*

In the early 1990s, when fluorescein angiography was the established modality for *in vivo* imaging of posterior pole haemodynamics, the terms ‘occult’ and ‘classic’ had been devised to describe the hyperfluorescence and leakage patterns, which were thought to correlate with neovascularization occurring in different tissue compartments and were also thought to account for differences in natural history and response to treatment in the macular photocoagulation studies.^20^, ^21^ Anatomically, neovascularization arising from the choroid can occupy the subretinal pigment epithelial space, the subretinal space or both, if it is to meet the criterion of ectopia, and it was along these lines that Gass proposed the type 1 (sub‐RPE) *vs.* type 2 (subretinal) anatomical classification of neovascularization, based on biomicroscopic and histological observations.^22^, ^23^ However, fluorescein angiograms could often be difficult to interpret unambiguously due to leakage effects and it was not until the advent of ICGA and, more importantly, OCT, that Gass’ classification could be applied in a clinically valid manner.^24^

### Depth‐resolved imaging

OCT was described in 1991 and its application to study the ocular posterior segment was first reported in the literature in 1995.^25^, ^26^ First generation time domain technology had limited axial resolution and depth penetration. The high reflectivity of the RPE posed a significant challenge for imaging more posterior structures relevant to the pathogenesis of PCV. Iijima *et al*. described the OCT contour of the RPE at the site of polypoidal lesions as ‘anteriorly protruding’ and subsequently contrasted the aspect ratio of polypoidal elevations of the RPE with that of serous PEDs.^27^, ^28^ Their studies constituted an attempt to characterize the nature of PCV from biomechanical inferences derived from RPE contour data.

In 2007, *Sato et al.* applied third‐generation OCT, with a superior axial resolution (≤10 μm), to study PEDs in 42 eyes with PCV. All eyes in their series exhibited peaked PEDs at the site of polypoidal lesions and all but one eye exhibited an adjacent irregular PED, correlated spatially to the angiographically detectable branching vascular network.^29^ Of the 41 PEDs, 17 were frank PEDs and 24 were shallow PEDs with a small but resolvable RPE–Bruch's membrane separation constituting a ‘double layer sign’ on OCT. Sato's group also noted that the contents of shallow irregular PEDs were usually at least intermediately reflective on OCT, putatively due to the vascular elements contained.

Visualization of the choroid and the lesion components of PCV was facilitated further by spectral domain OCT and enhanced depth imaging OCT, which provide levels of detail that correlate well with histology.^30^ Importantly, spectral domain OCT enables reliable visualization of Bruch's membrane as the posterior boundary of a PED cavity, allowing more precise analysis of the internal PED structure. Spaide observed that type 1 neovascular tissue in AMD is frequently adherent to the basal aspect of the elevated RPE, supporting Gass’ original theory that RPE tears seen in AMD resulted from shrinkage of neovascular complexes and tractional forces on the delicate RPE layer.^31^, ^32^

**Figure 2:**
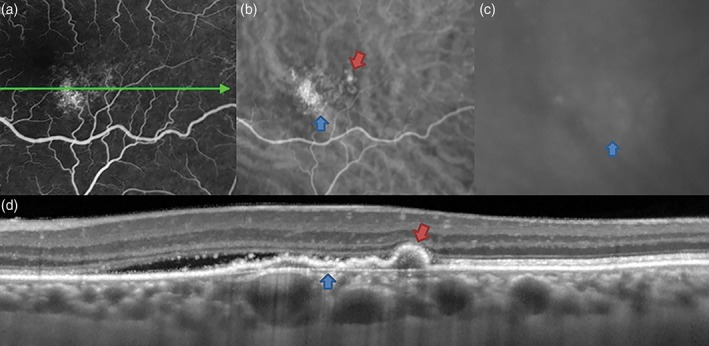
Aneurysmal type 1 neovascularization (polypoidal choroidal vasculopathy) in a 60‐year‐old white female. Mid‐phase fluorescein angiography (a) shows non‐specific hyperfluorescence inferotemporal to the fovea, consistent with ‘occult’ choroidal neovascularization. Mid‐ and late‐phase indocyanine green angiography findings (b, c) show a hyperfluorescent plaque (blue arrow) at this focus. In the transit phase, an aneurysmal (polypoidal) lesion is also noted (red arrow). Spectral domain optical coherence tomography (d) shows subretinal fluid adjacent to pigment epithelial detachment (PED). The PED has a shallow irregular component (blue arrow), corresponding to type 1 neovascularization, and a peaked component (red arrow) at the site of the aneurysmal (polypoidal) lesion. The choroid is relatively thick due to dilated Haller's layer vessels, anterior to which the inner choroid is attenuated.

Freund *et al*. used simultaneously acquired spectral domain OCT and ICGA to reinforce the implications of these findings as being relevant to the anatomical classification of neovascularization and described the localization of angiographically detected polypoidal lesions to the basal aspect of the RPE, convincingly *anterior* to Bruch's membrane (Fig. 2).^24^ This interpretation of the location of polypoidal lesions is compatible with the observations of Iijima *et al*., who described the peaked morphology of RPE elevation in polypoidal disease quantitatively.^28^ Freund *et al*. also advanced the case that polypoidal lesions arise from longstanding type 1 neovascular tissue and represent a component of that tissue, a view bolstered by the findings of Alshahrani *et al*.^33^ The role for OCT in the diagnosis of polypoidal disease was supported by De Salvo *et al*., who calculated favourable sensitivities and specificities of spectral domain OCT features for PCV.^34^

### AMD and polypoidal choroidal vasculopathy

**Figure 3:**
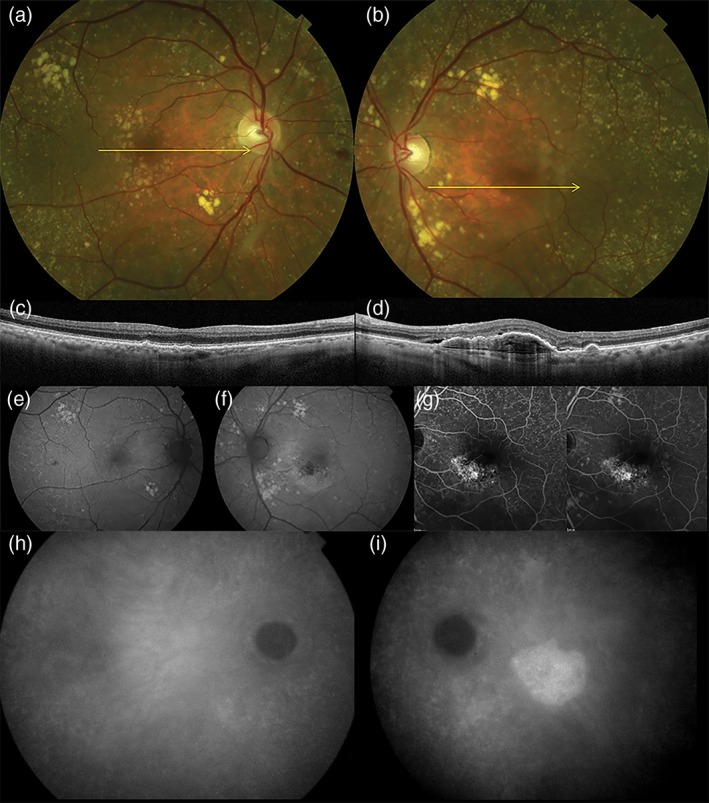
Age‐related macular degeneration in an 82‐year‐old white male. Colour photographs of the right and left eyes (a, b) show numerous drusen in the posterior pole and fundus tessellation in the macula. Spectral domain optical coherence tomography of the right and left eyes (c, d) shows bilateral drusen. In the left eye (d), there is a large irregular pigment epithelial detachment with hyperreflective contents and adjacent subretinal fluid, consistent with type 1 neovascularization. The choroid is relatively thin in both eyes. Fundus autofluorescence of the right and left eyes (e, f). There is a hypoautofluorescent geographic focus at the fovea of the left eye (f) with a curvilinear hyperautofluorescent zone *inferior* to the fovea, hinting at a legacy of chronic subretinal fluid. Late transit and recirculation fluorescein angiography of the left eye (g) shows irregular hyperfluorescence at the inferior border of the fovea with late, poorly defined staining. There is also extensive staining of drusen. Indocyanine green angiography of the right eye (g) shows no evidence of neovascularization. Indocyanine green angiography of the left eye (h) shows a hyperfluorescent ‘plaque’ indicating the full extent of the type 1 neovascular lesion.

**Figure 4:**
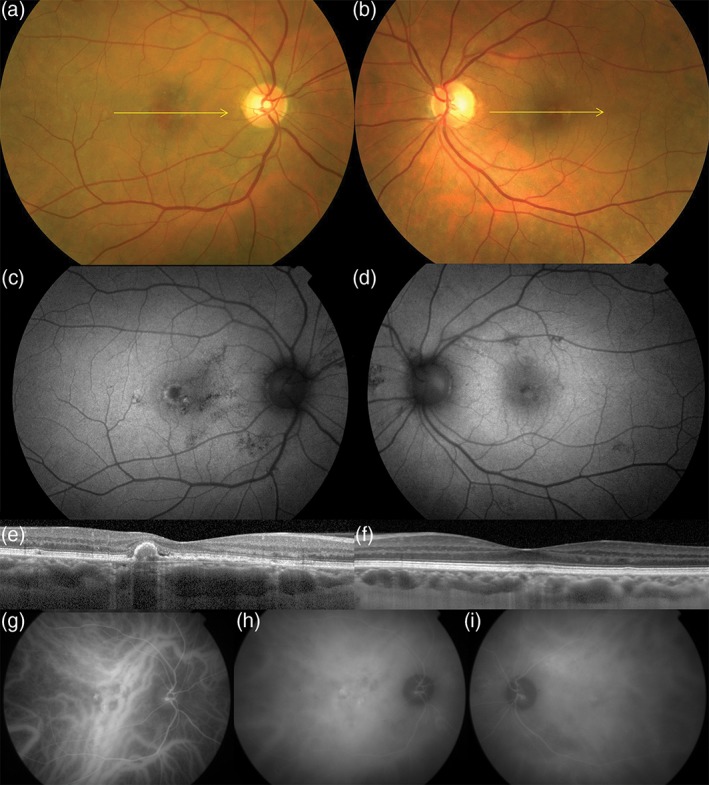
Aneurysmal type 1 neovascularization (polypoidal choroidal vasculopathy) in a 57‐year‐old white female. Colour photographs of the right (a) and left (b) eyes show absent drusen in the posterior poles. Fundus autofluorescence of the right (c) and left (d) eyes show hyper‐ and hypoautofluorescent changes in the macula and nasal to the optic nerves. Spectral domain optical coherence tomography of the right (e) and left (f) eyes. A shallow irregular pigment epithelial detachment is present in the right eye (e) with a peaked component, consistent with a type 1 neovascularization and an aneurysmal (polypoidal) lesion, respectively. Dilated Haller's layer veins are present in both eyes, especially so in the right eye (f) where, at the site of neovascularization, the inner choroid is attenuated. Early phase indocyanine green angiography of the right eye (g) shows pathologically dilated choroidal veins traversing the fovea with subtle hyperfluorescence of an overlying type 1 neovascular lesion (branching vascular network). A more intensely hyperfluorescent aneurysmal (polypoidal) lesion is seen at the temporal edge of the type 1 neovascular lesion. A hyperfluorescent plaque corresponding to type 1 neovascular lesion is seen in the mid‐phase (h). Areas of choroidal hyperpermeability are present nasal to the optic nerve in the right eye (h) and in the superotemporal macula of the left eye (i).

Although several clinical case variables suggest that PCV is *not* a form of AMD, the point continues to be debated.^35^ Important features which distinguish PCV from AMD include: (i) thicker subfoveal choroid than is typical for AMD; (ii) absence or paucity of drusen in PCV and, where present, drusen with an unusual shape^36^; (iii) predilection for PCV in pigmented individuals, particularly those of African descent in whom neovascular AMD is uncommon; (iv) the relatively young age of PCV cohorts; and (v) the development of polypoidal lesions. Figures 3 and 4 illustrate the multimodal findings in neovascular AMD and polypoidal disease, respectively.^37^ Overlap can certainly occur when individuals with polypoidal disease also exhibit age‐related macular drusen and type 1 neovascularization, particularly among elderly patients, but these factors in themselves do not necessarily unify the phenotypes into the same disease class.

It has been suggested that PCV is genetically related to neovascular AMD as patients with *either* are more likely to carry *ARMS2* susceptibility alleles than control subjects. Several single nucleotide polymorphisms conferring susceptibility to type 1 neovascularization (including an *ARMS2* allele, rs10490924) have indeed been shown to be shared between cohorts of patients with neovascular AMD and neovascular central serous chorioretinopathy, where each cohort was phenotyped diligently.^38^ This study also found a few patients with polypoidal lesions in a neovascular central serous chorioretinopathy cohort, but none in the neovascular AMD cohort. However, given the phenotypic differences between neovascular AMD and neovascular central serous chorioretinopathy, this finding raises the question as to whether certain alleles considered to confer risk for neovascular AMD might in fact be risk alleles for neovascularization in a more generic sense, as a final common pathway. Interestingly, in studying the role of *ARMS2* polymorphisms in PCV, Ma *et al*. found allelic diversity between neovascular AMD and PCV at the *ARMS2‐HTRA1* locus.^39^ Other authors have studied PCV genetics in detail and also found genetic heterogeneity that appears to have clinical relevance.^35^

### Pachychoroid

The term pachychoroid was introduced into the literature in 2013 by Warrow *et al*. to describe the occurrence of retinal pigment epitheliopathy in patients with choroidal findings resembling those of chronic central serous chorioretinopathy.^40^ These patients also exhibited reduced fundus tessellation at the posterior pole on ophthalmoscopy, choroidal hyperpermeability on ICGA, relatively thick subfoveal choroid and Haller's layer vessel dilation on enhanced depth imaging OCT, particularly at the sites of pigment epithelial hyperplasia. Some patients had these findings bilaterally. Pachychoroid pigment epitheliopathy was therefore described as a possible precursor, or *forme fruste*, of central serous chorioretinopathy as well as a phenotypic expansion.

The term pachychoroid neovasculopathy was defined by Pang and Freund to recognize that (i) type 1 neovascularization complicating central serous chorioretinopathy differed from that seen in AMD (reduced fundus tessellation, scarce drusen, thick hyperpermeable choroid and dilated Haller's layer vessels); (ii) type 1 neovascularization could arise in relatively young patients with pachychoroid neovasculopathy without antecedent neurosensory detachment attributable to central serous chorioretinopathy; and (iii) polypoidal lesions were overrepresented in pachychoroid neovasculopathy and appeared, on ICGA and OCT, to have arisen from sub‐RPE neovascular tissue.^41^ Patients meeting established criteria for a diagnosis of PCV had these pachychoroid features.

**Figure 5:**
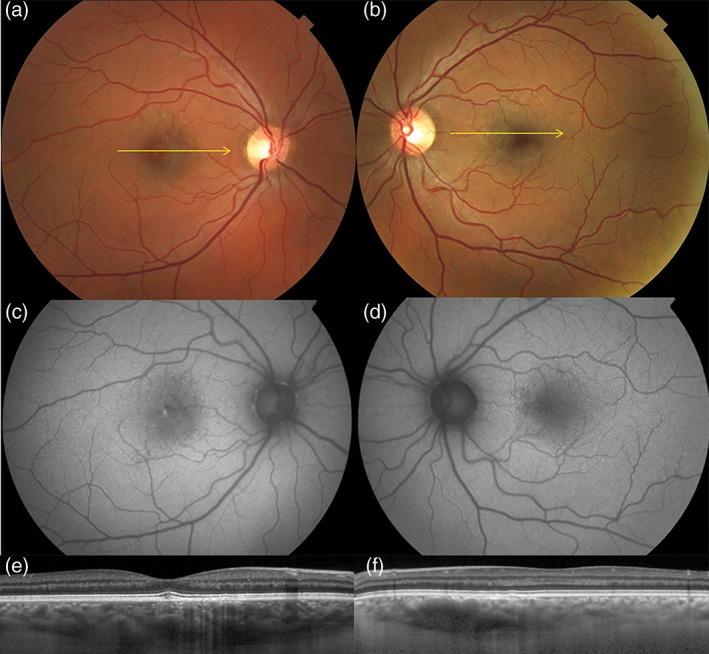
Pachychoroid pigment epitheliopathy in a 37‐year‐old white male. Colour photographs of the right (a) and left (b) eyes show absent drusen and reduced fundus tessellation. Fundus autofluorescence of the right (c) and left (d) eyes show non‐specific pigment epithelial changes, including a small focus of hyperautofluorescence at the right fovea (c). Spectral domain optical coherence tomography of the right (e) and left (f) eyes shows thick choroids in both eyes. In the left eye (f), a region of extrafoveal maximal choroidal thickness is attributable to Haller's layer vessel dilatation, anterior to which the inner choroid is attenuated.

Studies of pachychoroid eyes with *en face* swept source OCT consolidated the previous findings and emphasized the importance of inner choroidal thinning and relative Haller's layer thickening in identifying the pachychoroid mechanism, even in eyes in which the total choroidal thickness was not particularly high.^42^ Figure 5 illustrates structural choroidal features in a patient with pachychoroid pigment epitheliopathy.

Lee *et al*. examined a cohort of Asian patients with PCV to study these findings quantitatively. They found a *bimodal* distribution of choroidal thickness with peaks at 190 and 400 μm and a mean (±SD) subfoveal choroidal thickness of 268 ± 119 μm. In a more detailed analysis of a subset of patients with choroidal thickness <200 μm, they found an increased ratio of Haller layer thickness to total choroidal thickness, supporting the classification of PCV as a disorder driven by the pachychoroid mechanism, even when total choroidal thickness is relatively low. Koizumi *et al*. found that patients with PCV also exhibited choroidal hyperpermeability, co‐localized with polypoidal disease, and also interpreted their findings as supporting the resemblance to a central serous chorioretinopathy phenotype.^43^

### OCT angiography

A significant part of the debate over the true nature of PCV has been the *location* of the polypoidal lesions and of their feeding vessels. Much of the histopathological literature from the 2000s found fibrovascular tissue to be located close to or within Bruch's membrane, in some cases with an apparent *schisis* of Bruch's membrane on close examination of the presented figures.^13^ This relatively external location of the pathological tissue, together with the observation that in many cases of PCV the branching vascular network cannot be detected discretely by ICGA, led to the impression among many authors that PCV might be subclassified into at least two subentities, one with ‘polypoidal CNV’ and another with ‘PCV, an (intra‐)*choroidal* vasculopathy’. Accordingly, classification schemes have emerged using categories such as type 1 and type 2 PCV (completely separate ‘types’ from Gass’ neovascularization classification), or types A (interconnecting channels), B and C (branching vascular network on ICGA with or without leakage on fluorescein angiography, respectively).^44^ These classification schemes are difficult to follow and difficult to teach.

Optical coherence tomography angiography (OCTA) is a novel imaging technique that uses temporal and spectral characteristics from multiple structural OCT acquisitions to classify the probabilities of specific tissue locations as being dynamic as opposed to static. Advantages are that the resulting angiogram is depth resolved and that the final voxel intensity has a quantitative relationship to the flow data that is defined algorithmically. Disadvantages include the relatively small field of view and inability to detect permeability or leakage.

Spaide has used OCTA to study the anatomy of type 1 neovascular complexes in AMD and to discuss angiogenesis and the evolution of neovascular tissue in terms of thermodynamic and pharmacological principles.^45^ He has explained that the neovascular process is influenced not only by angiogenesis but also by pruning of small calibre channels as the neovascular tissue matures, which leads eventually to the vascular complex containing ‘trunk vessels’ which are well visualized by OCTA.

In the context of pachychoroid neovasculopathy, the neovascular process appears to be relatively insidious and indolent and it may be surmised that the dynamic balance between angiogenesis and pruning is shifted towards the latter, with implications for visualization by different angiographic techniques. Gaudric's group have studied shallow irregular PEDs in patients with central serous chorioretinopathy by conventional dye angiography and OCTA. They have reported detection rates for neovascularization on the order of 20% for dye angiography and 35% for OCTA.^46^, ^47^ In a cohort of patients with pachychoroid neovasculopathy, several of whom were treated for several years with antiangiogenic therapy and imaged with OCTA several years after the onset of RPE–Bruch's membrane separation, our group detected neovascularization in over 90% of study eyes, testifying to the predictive value of the shallow irregular PED seen on structural OCT as signifying the presence of type 1 neovascularization.^48^ Four of the 16 eyes in our study harboured polypoidal lesions. Similar findings have been reported in cases of quiescent, treatment‐naive type 1 neovascularization.^49^

**Figure 6:**
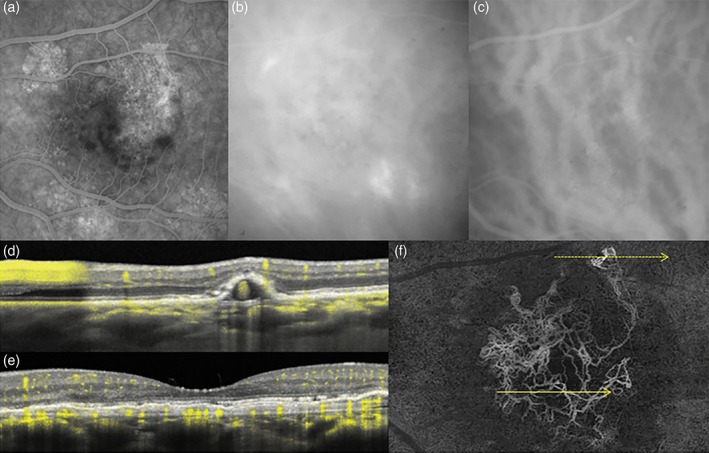
Fluorescein angiography (FA), indocyanine green angiography (ICGA) and optical coherence tomography (OCT) angiography of the left eye in 59‐year‐old white male with aneurysmal type 1 neovascularization (polypoidal disease). Non‐specific hyperfluorescence is seen on FA (a) while ICGA (b, c) shows dilated Haller's layer veins, choroidal hyperpermeability, and a focal area of hyperfluorescence (arrow). Dense raster cross‐sectional OCT (d, e) shows a shallow irregular pigment epithelial detachment (PED) in the central macula and a peaked extrafoveal PED, consistent with aneurysmal type 1 neovascularization (polypoidal disease with a branching vascular network). The angiographic signal overlay (yellow pixels) confirms flow within the shallow PED (e) and the aneurysmal lesion (g). *En face* swept source OCT angiography (f) segmented immediately anterior to Bruch's membrane reveals the morphology of the type 1 neovascular network, the distant location of the aneurysmal lesion and its feeding vessels originating from the neovascular tissue. The direction and location of the dotted and yellow arrows correspond to (d) (upper arrow) and (e) (lower arrow) cross‐sectional scans, respectively.

These OCTA findings raise the question as to whether the subtypes into which some authors seek to classify PCV—based on the presence or absence of branching vascular networks and ‘plaques’^50^—may actually represent different *stages* of disease at the time of diagnosis, possibly with different underlying speeds of angiogenesis, pruning and aneurysmal change. The OCTA data have shown that even polypoidal lesions which have arisen in apparent isolation are frequently fed by vessels coursing tangentially through shallow irregular PEDs over relatively long distances (Fig. 6).^48^, ^51^ Without the depth resolution of OCTA, such vessels may be difficult to identify as (type 1) neovascularization because they are large in calibre, pruned, non‐permeable and do not form ‘plaques’ in late phase ICGA (e.g. fig. 4 in Ref 52).

### Aneurysms *versus* polyps

Aneurysms are focal dilatations of blood vessels and occur due to localized weakness of the vessel wall. The lumen of an aneurysm may contain blood, fibrin or thrombus; may experience low or turbulent flow and is generally pressurized together with the main vessel. Aneurysms on vessels of all sizes have the potential to rupture and bleed. In microvascular networks, aneurysms may be *permeable* to transudation or exudation. Polyps are fleshy masses of tissue which arise from epithelial surfaces, usually from mucous membranes. Polyps are typically not vascularized unless they grow substantially to induce angiogenesis within their peduncles and cores.

The characteristic lesions of PCV were described as polypoidal (adjective) because although PCV was recognized as a vasculopathy, the shapes of the lesions were reminiscent, at least in outline or silhouette, of the shapes of polyps. Moreover, the precise origins and composition of these lesions had yet to be determined.

The terms ‘aneurysmal’ and ‘saccular’ are well established in the medical nomenclature and appear throughout the PCV literature starting from the very first publication by Yannuzzi *et al*.^1^, ^15^ In light of the accumulated knowledge on PCV over the past three decades, which has confirmed and reinforced the understanding of PCV lesions as a form of aneurysm, returning to this basic terminology brings back into perspective the fundamental questions to be answered if the aetiology of these lesions is to be elucidated. Once PCV is understood as ‘aneurysmal type 1 neovascularization’, the question arises as to why some type 1 neovascular complexes form aneurysms while others do not.

Aneurysms occur when a focal weakness in a vessel wall causes elastic decompensation. Such weakness can be attributable to factors such as atherosclerosis, vessel wall atrophy, collagen vascular disease, inflammatory disease, radiation,^53^, ^54^ genetic factors, trauma and, in capillaries, glycosylation and pericyte loss. Also essential to aneurysm formation is luminal pressure to drive the dilatation, making hypertension,^55^, ^56^ shunting and venous stasis potential contributing factors. The observations that aneurysmal type 1 neovascular networks have shown hyalinization in several histopathological studies and possibly some relationship with posterior ciliary arteries^13^ suggest that these networks might be subject to higher blood pressures and flows than those seen in non‐aneurysmal neovascularization (e.g. in AMD), a conjecture which might be validated by a deeper understanding of the changes in flow dynamics induced by the pachychoroid mechanism (manuscript under preparation).

## Conclusion

Aneurysmal type 1 neovascularization/PCV was originally described as a peripapillary choroidal disease in middle‐aged females, mainly of African ancestry. Since then, the demographic has been expanded to include Asian patients, so that the PCV bibliography is now dominated by papers from the Far East and Southeast Asia. It is also recognized that PCV can occur with moderate incidence in white populations.^57^, ^58^ In each of these groups, there is mounting evidence that the mechanisms that underlie aneurysm formation differ from those which promote neovascularization in AMD. In addition, it has been recognized that aneurysmal type 1 neovascular lesions can occur in contexts far removed from either AMD or typical PCV, examples being choroidal nevi, myopic staphylomata and dome‐shaped maculopathy.^59^, ^60^, ^61^

Previous reviews and editorials on polypoidal disease have sought to expand the taxonomic hierarchy to reconcile the diagnostic criteria for PCV with the expanding set of findings from multimodal imaging and the expanding set of contexts within which aneurysmal lesions are recognized.^35^, ^37^, ^62^ The result is an increasingly complex schema which becomes multiclass, highly nuanced and increasingly unwieldy, with a growing number of scenarios which do not quite fit and which are consigned to a category ‘other’.

In the present study, we have taken a step back towards the root of the taxonomy, so as to plot our position in the ‘bigger picture’ and pose questions and propose avenues for investigation which diverge from the mainstream literature but which might guide investigative efforts in a conceptual rather than factual direction. The main lesson to be learned is that, as a phenotypic spectrum appears to merit expansion, investigators should pause to consider the possibility that the entity being described is *not* a disease but a manifestation of many.
